# Seasonal coastal residency and large-scale migration of two grey mullet species in temperate European waters

**DOI:** 10.1186/s40462-024-00528-z

**Published:** 2025-01-11

**Authors:** Jena E. Edwards, Anthonie D. Buijse, Hendrik V. Winter, Allert I. Bijleveld

**Affiliations:** 1 NIOZ Royal Netherlands Institute for Sea Research, Den Burg, The Netherlands; 2 Wageningen University and Research, Wageningen, The Netherlands; 3 Deltares, Delft, The Netherlands; 4 Wageningen Marine Research, Wageningen University & Research, IJmuiden, The Netherlands

**Keywords:** Acoustic telemetry, Animal migration, Coastal ecosystem, Movement ecology, *Mugilidae*, Dutch Wadden Sea, North Sea

## Abstract

Grey mullets (family *Mugilidae*) are widespread across coastal, brackish, and freshwater habitats, and have supported fisheries for millennia. Despite their global distribution and commercial value, little is known about their movement ecology and its role in the co-existence of sympatric mullet species. Gaps in knowledge about migratory behaviour, seasonal occurrence, and movement scales have also impeded effective management, highlighting the need for further research. This study aimed to identify key habitats and timing of grey mullet presence across the Dutch Wadden Sea, North Sea, and freshwater areas, and to explore potential behavioral differences between two grey mullet species: thicklip mullet (*Chelon labrosus*) and thinlip mullet (*Chelon ramada*). Using acoustic telemetry, we tracked 86 tagged grey mullet over three years (thicklip mullet*,* N = 74*;* thinlip mullet*,* N = 12), combining data from 100 local acoustic receivers and the European Tracking Network. Both species were detected in the Wadden Sea from April to November, however, thinlip mullet arrived in the Wadden Sea earlier than thicklip mullet (median date = May 16 *vs.* June 7). Individual residency in the Wadden Sea lasted a median 97 days for thicklip mullet and 94 days for thinlip mullet. Thinlip mullet were also detected by more receivers and over a larger area than thicklip mullet, indicating differences in movement behaviour. Both species showed an affinity for receivers near major harbours, with thinlip mullet more often detected near fresh water outflows. Seasonal migrations between coastal and offshore waters were also observed, with one thinlip mullet returning to freshwater across consecutive years. North Sea detections spanned ten months, with a gap during the presumed spawning period (Jan–Feb). Our data suggest that thinlip mullet show a preference for deeper gullies while thicklip mullet may spend more time in shallow areas and flooded tidal flats. These findings highlight the importance of the Wadden Sea as a seasonal foraging ground and provide insights into the migratory patterns of grey mullets.

**Supplementary Information:**

The online version contains supplementary material available at 10.1186/s40462-024-00528-z.

## Background

Differences in movement ecology can promote niche separation and reduce competition among species that coexist in the same geographic area—a phenomenon known as sympatry [23, 24, 47, 49]. Sympatry is common among grey mullets (Mugilidae), a group of bony fishes that play a dominant role in coastal and estuarine waters across temperate, tropical, and subtropical latitudes in both hemispheres [27]. This family is comprised of 71 species worldwide, with 8 species occurring in European waters of the Northeast Atlantic, Mediterranean, and Black Sea [50]. In much of the world, grey mullets support commercial, artisanal and subsistence fisheries, and are cultured in both intensive and extensive aquaculture systems. Despite this broad cultural and economic significance, ecological knowledge of grey mullets remains limited, with research on wild populations confined to a relatively small proportion of their total range.

Grey mullets play a specialised ecological role in marine, brackish, and freshwater food webs due to their unique dependence on detrital material and in particular, microphytobenthos—a food source exploited by no other fishes to such a great extent [27, 55, 65]. Although grey mullets are remarkably similar in external appearance, they vary widely in their internal anatomy, environmental tolerances, and life history strategies [27]. As a group, mullets are highly flexible, with each species suited to a particular set of aquatic conditions ranging from extremely clear, to highly turbid waters, and individuals capable of adapting to a wide range of salinities [27, 43]. In some areas, multiple grey mullet species coexist, despite occupying highly similar dietary niches [10]. Previous studies have cited the importance of both trophic niche breadth and differences in osmoregulatory capacity in structuring grey mullet communities [20, 22] with salinity-driven differences in distribution patterns observed both in the aquaculture settings and in the wild [19, 21, 43]. The migratory patterns of adult grey mullets also vary both regionally and among species. In the Mediterranean, year-round estuarine residency has been observed [15], while in more northern regions such as Portugal, France, the UK, and the Baltic Sea, a catadromous lifestyle predominates, marked by seasonal migrations between freshwater and estuarine habitats and the coastal marine realm [40, 66, 78]. During summer, adult mullets in these northern areas concentrate in estuaries, brackish lagoons, and freshwater habitats in summer to feed [2, 40, 55, 78]. Then in autumn, decreasing photoperiod and temperature trigger downstream migrations into estuaries or coastal marine waters for spawning [40, 51, 55].

Overall, detailed knowledge of the distribution and movement ecology of European grey mullets remains limited, particularly in marine environments, where few telemetry studies have been conducted in only a few discrete locations [2, 5, 44]. The Wadden Sea, the world’s largest intertidal area, spanning the coastlines of the Netherlands, Germany, and Denmark, is home to three sympatric grey mullet species: the thicklip grey mullet (*Chelon labrosus*, hereafter, thicklip mullet), thinlip grey mullet (*Chelon ramada,* formerly *Liza ramada*, hereafter, thinlip mullet), and golden grey mullet (*Chelon auratus*). Among these, the thicklip mullet is the most common in this region but has faced dramatic local declines since the 1980s, with little evidence of recovery [36, 45, 73]. Although small-scale fyke and gill net fisheries targeting grey mullets still exist in the Wadden Sea, reduced catches have led many commercial fishers to desert the practice entirely. For those fisheries that remain, grey mullets are targeted in shallow areas close to the shoreline where individuals are known to aggregate while grazing on algae and benthic prey [56]. Due to their limited catchability in typical survey gears (beam trawls, stow nets, and fykes), grey mullets have also been underrepresented in fisheries surveys in the Wadden Sea [68]. As a result, much of the knowledge of their distribution is derived from historical records [11, 12] and the experience of local fishers, primarily catching the then-dominant thicklip mullet. Recent studies have mainly explored the diets and trophic ecology of grey mullets in the Wadden Sea [56, 57], leaving many movement-related questions unanswered.

Given the observed declines in the Wadden Sea and the need to develop effective management strategies, additional studies are essential for understanding the large-scale movements and seasonal habitat use of local grey mullets [36]. Similar to other northern habitats, grey mullet presence in the Wadden Sea varies seasonally, with adult catches peaking in early spring as migrants enter coastal waters and declining through autumn, suggesting the gradual departure of offshore migrants throughout the summer [11, 45]. However, whether all grey mullet species display similar spatial and temporal movement behaviours in this region remains to be seen. Evidence from other European habitats indicates differences in physiology and habitat use among grey mullets, underscoring the need for research into how movement patterns and space use differ within this diverse taxonomic group [21, 40, 43]. For example, it is unknown whether spatial or temporal segregation is present among sympatric mullets in the Wadden Sea and how this could impact the effectiveness of targeted management actions.

This study uses acoustic telemetry to investigate the movement patterns of two sympatric grey mullet species—the thicklip mullet and thinlip mullet—within and beyond boundaries of the Dutch Wadden Sea. Specifically, we aim to provide foundational insights into how grey mullets use this coastal region, with a focus on its role in their full migratory cycle and the identification of species-specific differences in movement behaviour. At a regional scale, we contextualise the Wadden Sea within the migration cycle by examining connections to adjacent habitats in the southern North Sea and fresh water. We then narrow our focus to the western Dutch Wadden Sea, where we aim to define the characteristics of habitat use for each species while disentangling differences in the timing of arrival and departure, individual movement trajectories, and overall patterns of mobility and space use.

## Methods

### Study area

The Wadden Sea is a dynamic coastal habitat with nearly 50% of its surface area covered by intertidal mudflats and the remainder composed of subtidal gullies connected to the North Sea [29]. This area encompasses diverse fish habitats including salt marshes, shallow littoral zones, and deeper sublittoral areas with soft sediment and occasional hard substrates such as oyster and mussel beds [7]. Compared to the adjacent North Sea, the Wadden Sea is rich in suspended organic matter and silt, creating a highly productive, yet turbid environment [58].

**Fig. 1:**
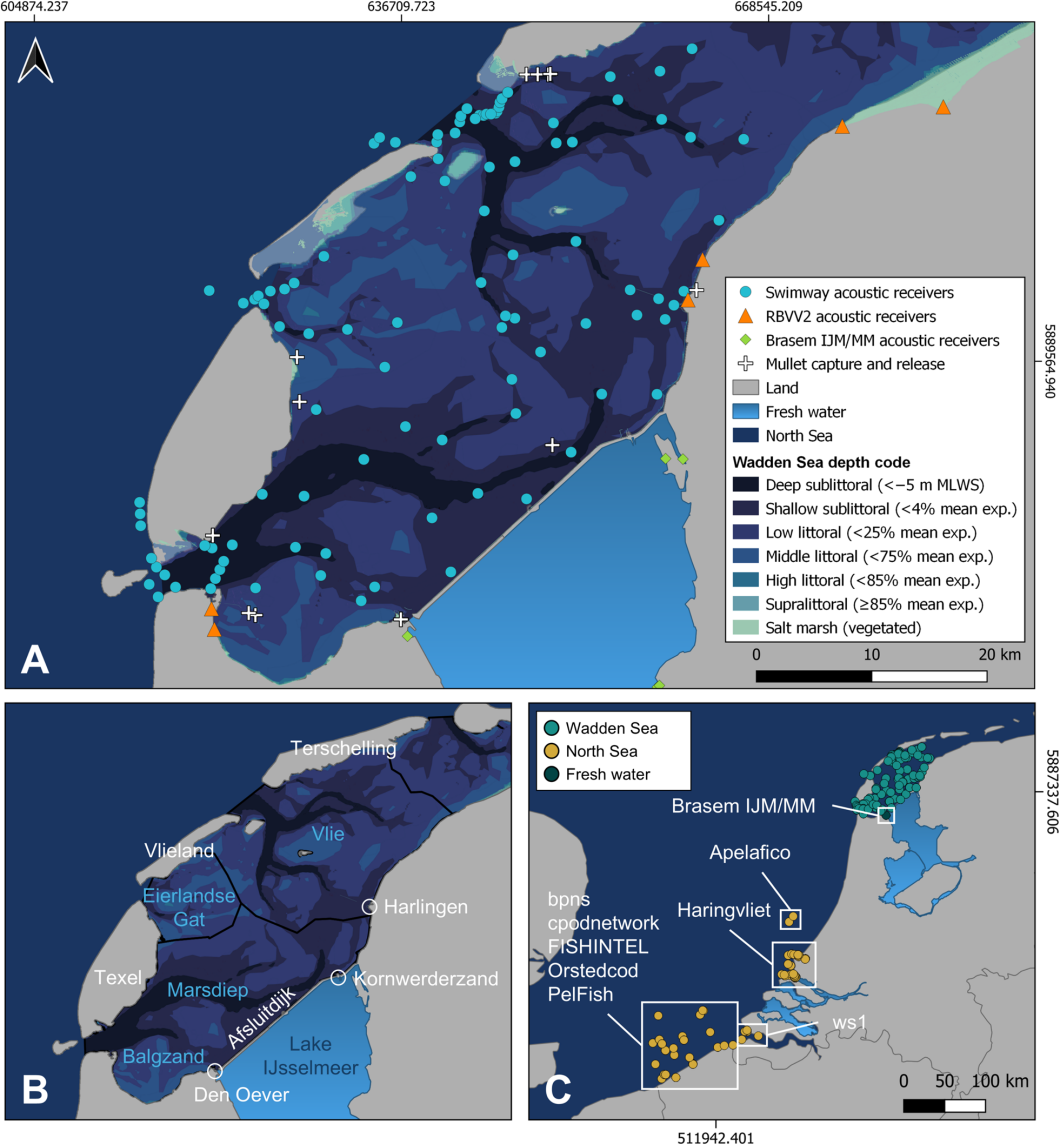
Locations of acoustic receiver stations in **A** the western Dutch Wadden Sea and northern Lake IJsselmeer and **C** the southern North Sea. Depth contours of the Wadden Sea indicate the upper limits of zones described by [7], ranging from areas exposed at low tide (salt marsh to low littoral) to deep sublittoral areas that are permanently below mean low water spring tide (MLWS) and reach maximum of ~ 40 m depth. **B** Notable locations referred to in the text, including three tidal basins (blue text) and one subregion (Balgzand). Acoustic receiver locations in panels **A** and **C** encompass multiple acoustic arrays: i) Wadden Sea—SWIMWAY, RBVV2, ii) North Sea—Apelafico, bpns, cpodnetwork, FISHINTEL, Haringvliet, Orstedcod, PelFish, ws1, and iii) Fresh water—Brasem IJM/MM. Only receivers with mullet detections are included in panel **C**. Additional detection data were obtained via the database of the European Tracking Network: https://www.lifewatch.be/etn/

The western Dutch Wadden Sea, bordered by the Dutch mainland and a chain of barrier islands, has an average depth of only 3.5 m [85] and covers an area of ~ 1530 km^2^ (Fig. 1). It contains three tidal basins—Marsdiep, Eierlandse Gat, and Vlie—separated by tidal divides where flood waters from adjacent inlets converge [7] (Fig. 1b). To the south, fresh water inputs from mainland sluices and the Afsluitdijk—a large barrier built in 1932 to separate the Wadden Sea and lake Ijsselmeer—drive broad-scale salinity gradients extending throughout the region [58].

Historically, the Wadden Sea has been a key nursery and feeding ground for various fish species, particularly those that spawn in deeper waters of the North Sea and English Channel [73, 74]. However, a combination of global warming and human activities, including fisheries, dredging, and resource extraction, have degraded fish habitats in both the Wadden and North Seas, contributing to population declines over the past four decades [69, 73]. Furthermore, sea surface temperatures in the Dutch Wadden Sea have increased by 1.5 °C over the last 25 years [71], with potential consequences for fish migration and distribution [77].

### Fish tagging

Grey mullets were captured using gillnets throughout the coastal waters of the western Dutch Wadden Sea (see Fig. 1 for capture locations). Additional captures were obtained from a long-term fyke net survey conducted in the Marsdiep Channel [72].

Upon capture, individuals were sedated via submergence in an anaesthetic bath containing a solution of 2-phenoxyethanol (0.04%) and seawater. Once sedated, fish were removed from the anaesthetic and measurements (total length; L_T_) and biological samples (2 fin clips, 3–4 scales) were taken. Each fish was externally marked with a Floy tag anchored into the dorsal musculature approximately 1 cm ventral to the second dorsal fin. Acoustic transmitters (V13-1x, V13AP-1x, V16-4x; Innovasea Systems Inc., Bedford, Canada) were then implanted in the intracoelomic cavity using a small incision on the ventral side, anterior to the anal fins, and closed with 2–3 dissolvable polyethylene sutures (for tag specifications see Table S1, Supplementary Materials). Tagging was performed in accordance with the Experiments on Animals Act in the Netherlands and approved by the Animal Ethical Commission under permit numbers 2016.D-0041 and 2021.D-0002 (Wageningen Marine Research).

In the spring and summer months of 2021 (May 12–July 1) and 2022 (May 6–Sept 14), a total of 125 grey mullet were captured in the western Dutch Wadden Sea (Fig. 1) and equipped with acoustic transmitters. This group was comprised of 106 thicklip mullet, 16 thinlip mullet, and 3 golden grey mullet (Table 1). As only a single golden grey mullet was detected, the remainder of this study focuses on thicklip and thinlip mullet.

**Table 1:** Tagging data for grey mullets (genus *Chelon*) tagged in the western Dutch Wadden Sea

Species	N tagged	N detected	Capture regions	Median length (cm)	Length range (cm)	V13AP	V13	V16
Thicklip	106	74	Balgzand, Eierlandse Gat, Texel, Terschelling	51.5	42.8–69.5	20	79	7
Thinlip	16	12	Den Oever, Harlingen, Kornwerderzand	54.3	40.7–61.2	0	12	5

Detected fish are those remaining in the filtered dataset

V13AP, V13, and V16 refer to transmitter models from Innovasea Systems Inc. (Bedford, Canada), and are listed with numbers deployed

### Acoustic arrays

This study incorporates detection data from multiple receiver arrays in the Wadden Sea and southern North Sea, sourced from the European Tracking Network data portal (https://www.lifewatch.be/etn/) developed by the Flanders Marine Institute as part of the Flemish contribution to LifeWatch (see Appendix 1 for individual contributors and array acknowledgements). Receiver stations that detected mullet were classified according to three regions. First, the Wadden Sea was defined as being within the boundaries of the western Dutch Wadden Sea and included detections from the projects SWIMWAY [34] and RBVV2 [41] (Fig. 1a). Mullet detections in fully fresh water were recorded by a single receiver from the project Brasem IJM/MM [75] in Lake IJsselmeer (Fig. 1a). Lastly, the North Sea included any receivers outside the Wadden Sea in the Dutch coastal zone—Apelafico [30] and Haringvliet [48], Western Scheldt—ws1 [62], and in coastal and offshore Belgian waters—bpns [61], cpodnetwork [32], FISHINTEL [67], Orstedcod [82], and PelFish [31] (Fig. 1c).

In the western Dutch Wadden Sea, this study relied primarily on the SWIMWAY array, comprised of 81–100 receiver stations per 6-month deployment period (median = 93) spanning from the Marsdiep Channel in the west to the eastern edge of the Vlie basin (1529 km^2^, Fig. 1a). Receivers were checked and data downloaded every 6 months due to strong currents in much of the array which had the potential to result in receiver loss without frequent maintenance. The number of active receivers varied throughout the study period due to receiver damage, loss, and replacement. Receivers were deployed via attachment to floating navigational buoys situated in the subtidal gullies and tidal inlets across three tidal basins (for detailed description and diagrams see [35]. All receivers were attached within 1–2 m of the sea surface with the hydrophone oriented toward the seabed. Average midpoint detection range (149 m high power transmissions [152 dB], 310 m very high power [160 dB]) and maximum range (610 m high power, 890 m very high power) were based on range tests conducted in the first year of study [35].

### Data analysis

Acoustic detection data were analysed using R software [59] and the packages *tidyverse* [81], *sp* [9, 54], and *sf* [53]. Plots were created with *ggplot2* [80] and *ggspatial* [33].

Prior to data analysis, false detections were removed from the dataset using a series of filters [14]. First, we removed all detections occurring prior to tag deployment, duplicate detections (*i.e.*, detections occurring at precisely the same time for a single transmitter), and detections occurring within an interval smaller than the tag’s minimum transmission delay (Table S1, Supplementary Materials). Single detections occurring at one receiver station within a 24 h period were also classified as unreliable and were removed. Detections were removed from the dataset for individuals identified as having died during the study period, either through recovery of internal tags or indications from examination of individual detection profiles (N = 9; see Supplementary Materials for further details). Data from 2024 are shown in data summaries but are not included in statistical analyses due to limited sample size (only one fish detected).

### Connectivity across aquatic habitats

To reveal broad-scale spatial and temporal patterns in fish presence over the full study area, acoustic detections were summarised by array across three aquatic regions: the Wadden Sea, North Sea, and fresh water. Movements connecting receivers across these regions were categorized based on their direction relative to the Wadden Sea and visualized for both species.

### Patterns of occurrence in the Dutch Wadden Sea

Detections of grey mullets in the Wadden Sea were visually examined to identify potential hotpots of detection for each species. Visualisations include receiver stations with no detections, serving as a proxy for fish absence or areas of reduced detection efficiency. Receiver stations that incurred damage during the deployment period or had deployment periods of < 90 d (half of the standard 6-month period) were excluded. Individual detection profiles were also visually examined to highlight variations in movement behaviour.

### Timing and duration of Wadden Sea residence

Arrival and departure times for each tagged fish were based on the dates of first and last detection on Wadden Sea receivers (Swimway or RBVV2 arrays) in each year. As individuals were already present at tagging, arrival and departure timing was estimated only for years following tagging, referred to as ‘return years’. Residence duration, defined as the time between first and last detection, was also calculated only for return years. To minimize the inclusion of partial residence periods,—*e.g.,* during which a fish was detected only while entering or leaving the array—only individuals with residence durations > 5 days were included in the analysis. Median residence durations, arrival, and departure dates were compared between species and years using Kruskal–Wallis rank-sum tests, suitable for non-normally distributed data.

A residency index (RI) was calculated for each fish to examine differences in detectability between the two species, highlighting potential variations in movement behaviour or habitat use during the period of Wadden Sea residence. RI was calculated as the number of days a fish was detected in the Wadden Sea divided by the length of the residence duration [4, 25, 42].

$$\text{RI}= \frac{N \text{detection days}}{\text{Residence duration }(\text{days})}$$

Values close to 1 indicate frequent detection throughout the residence period, implying the use of areas with high receiver coverage. In contrast, values near 0 suggest reduced detection rates, possibly due to the use of areas outside of detection range. When combined with the number of stations by which an individual was detected, RI also serves as a proxy for relative mobility. High RI values associated with high receiver counts may indicate extensive movement within the array, while high RI and low receiver counts suggest prolonged residency near specific receiver stations. Low RI values may reflect the use of areas outside of receiver coverage or limited residency within the array area. As such, RI can highlight inefficiencies in array design in arrays with uneven receiver distribution (such as the SWIMWAY array, Fig. 1a). Given the numerous biological and methodological factors involved, RI values should be interpreted with care [4].

### Space use in the Wadden Sea

Three metrics of space use were used to assess the spatial scale of movements exhibited by tagged individuals during their seasonal residence period in the Wadden Sea. We first determined the number of unique receiver stations by which each fish was detected. Station counts were used as a proxy for mobility, where higher station counts suggested greater mobility.

Next, the maximum extent of movements within the array area was estimated by calculating a Minimum Convex Polygon (MCP) for each individual and tracking year as a proxy for the extent of individual space use during the summer residence period. This was done by tracing the smallest possible polygon around the receiver stations used, connecting the outermost stations with interior angles less than 180° [42]. Calculated areas were corrected to remove overlap with land and reduce overestimation. It should be noted that this method is a coarse estimate of space use that is dictated by array design and assumes complete use of all areas within the polygon. For our purposes, MCP area was used to estimate the extent of individual movements within the area of receiver coverage in the western Dutch Wadden Sea.

Lastly, a local residency index (RI_*stat*_) was used to quantify the proportion of detections recorded at each receiver station for each tagged fish [4]. This was calculated by dividing the number of days a fish was detected at a specific station in a given year by the total number of days it was detected across the entire array. This provides insight into the relative importance of each receiver station in detecting individual fish.

$${\text{RI}}_{stat}= \frac{N \text{days detected at unique receiver station}}{N \text{days detected in array}}$$

For each receiver station, the median RI_*stat*_ value was calculated across all tagged fish and study years. A visual examination of mean RI_*stat*_ values for all stations was then used to compare the distribution of fish presence across the array for each species. Lower values suggest that unique receiver stations provided a small proportion of total detections, acting as an indicator of high mobility within the array.

For all three metrics of space use, Kruskal–Wallis rank sum tests for non-normal data were used to detect differences between species and between years within each species group. In cases of significance, a Dunn post hoc test with a Bonferroni correction was performed to further investigate the differences between groups.

## Results

### Connectivity across aquatic habitats

Of 122 tagged thicklip and thinlip mullet, 86 individuals were detected over the three-year study period (May 12, 2021 to June 23, 2024) resulting in a filtered dataset containing 21,146 detections (Table 2). Detected individuals included 74 thicklip and 12 thinlip mullet. Detections were recorded across three distinct regions: the western Dutch Wadden Sea (85 individuals, 13,018 detections), the southern North Sea (29 individuals, 7,751 detections), and the fresh waters of Lake IJsselmeer (1 individual, 377 detections) (Table 2).

**Table 2:** Summary of acoustic detections for thicklip and thinlip mullet (N = 74, N = 12) tagged in the Dutch Wadden Sea and detected between May 2021 and June 2024

Array name	Region	Distance (km)	N fish	N stations	N detections	N Years	Months
SWIMWAY	Wadden Sea	0	85	86	9652	4	April–Nov
RBVV2	Wadden Sea	7	13	4	3366	2	May–Oct
bpns	North Sea	250	13	14	414	3	March–June, Aug–Dec
Haringvliet	North Sea	153	8	28	7225	2	April–Nov
cpodnetwork	North Sea	254	8	5	49	2	Aug–Oct, Dec
ws1	North Sea	215	5	5	29	3	April–May, Aug, Oct
Apelafico	North Sea	165	3	3	10	2	Aug–Oct
FISHINTEL	North Sea	259	3	4	17	2	April, Oct, Dec
Orstedcod	North Sea	219	2	2	4	2	Oct
Brasem_IJM/MM	Fresh water	23	1	1	377	2	June–July
PelFish	North Sea	261	1	1	3	1	May

Distances refer to the distance between array center points relative to the SWIMWAY array

N stations refers to the total number of stations in each array that detected tagged fish. Excluding data from the SWIMWAY and Haringvliet arrays, detection data were obtained via the database of the European Tracking Network: https://www.lifewatch.be/etn/

**Fig. 2:**
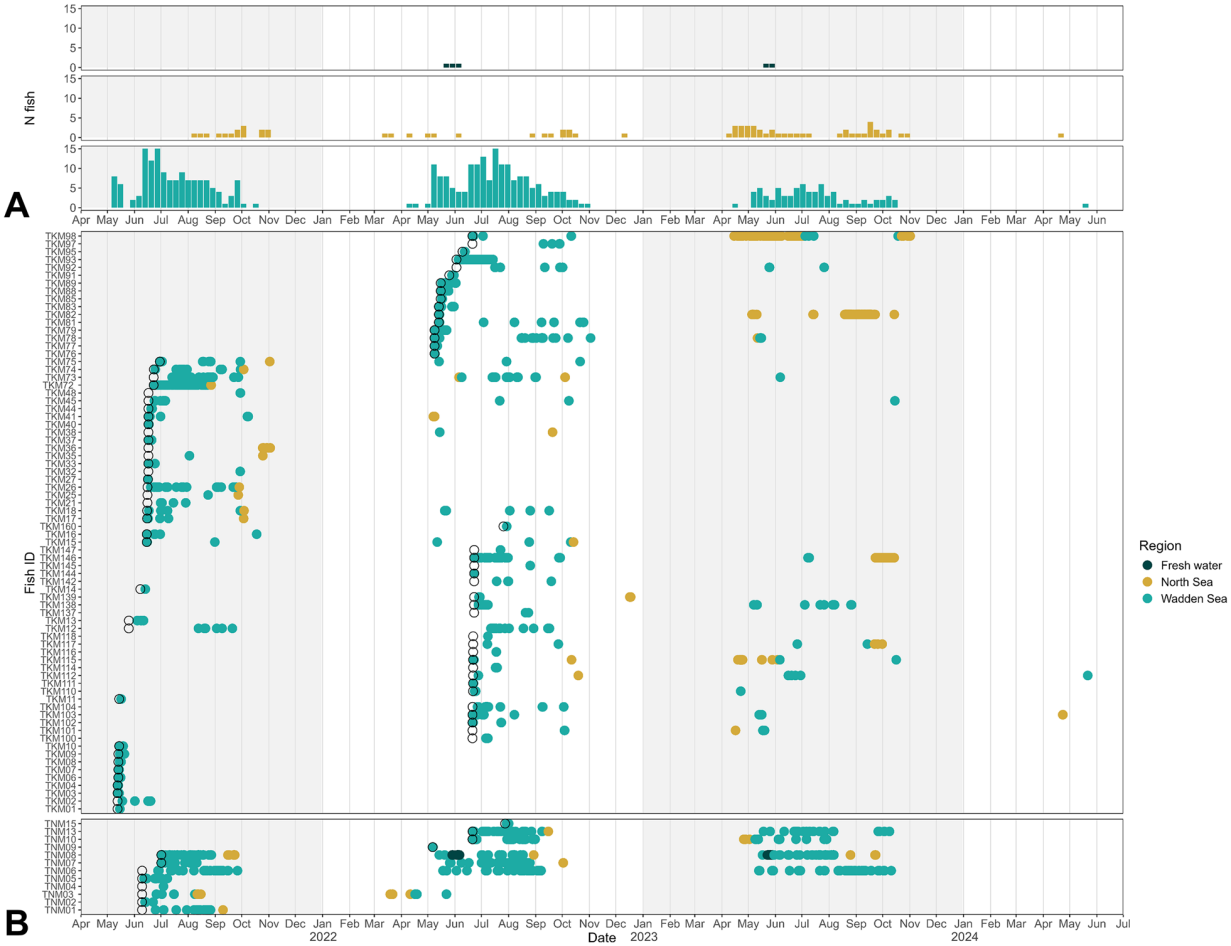
Time and locations of acoustic detections for thicklip mullet (TKM) and thinlip mullet (TNM) tagged and detected in the western Dutch Wadden Sea between May 2021 and June 2024. **A** Number of unique fish detected per week in three regions. **B** Individual detection profiles coloured by region with open circles indicating the date of tagging and release. All transmitter lifespans extend beyond the range of this figure

Fish presence varied seasonally across the Wadden Sea, North Sea, and freshwater regions. In the Wadden Sea, detections occurred annually from spring to autumn (April-November), with occasional forays into fresh water in May and June (Fig. 2). A total of 73 thicklip and 12 thinlip mullet were detected by 90 unique receiver stations in the western Dutch Wadden Sea between 2021 and 2024. Most individuals were detected for only one year (N = 61), while others were redetected over two (N = 19) or three consecutive years (N = 5) (Fig. 2b).

Fish presence in the North Sea spanned ten months of the year (March-Dec), showing overlap in timing with fish presence in the Wadden Sea (Fig. 2). In this region, 24 thicklip and 6 thinlip mullet were detected by 61 unique receiver stations deployed in the Dutch coastal zone, the Western Scheldt, and in Belgian waters (Table 2, Fig. 1c). Detections were recorded year-round, except in January and February, with peaks in fish presence recorded in May (May, median = 3, IQR = 2–4) and again in autumn (September and October, median 4–5 fish per month). Notably, 93% of North Sea detections were recorded by the Haringvliet array, located at an outflow of the Rhine-Meuse delta (153 km from the Wadden Sea’s centre). Detections in this area overlapped with periods of fish presence in the Wadden Sea and were associated with similar durations of individual residence (Fig. S2, Supplementary Materials). Meanwhile, one array off the Belgian coast (bpns, 219–260 km from Wadden Sea) recorded far fewer detections, but more unique individuals (N = 13) (Table 2).

**Fig. 3:**
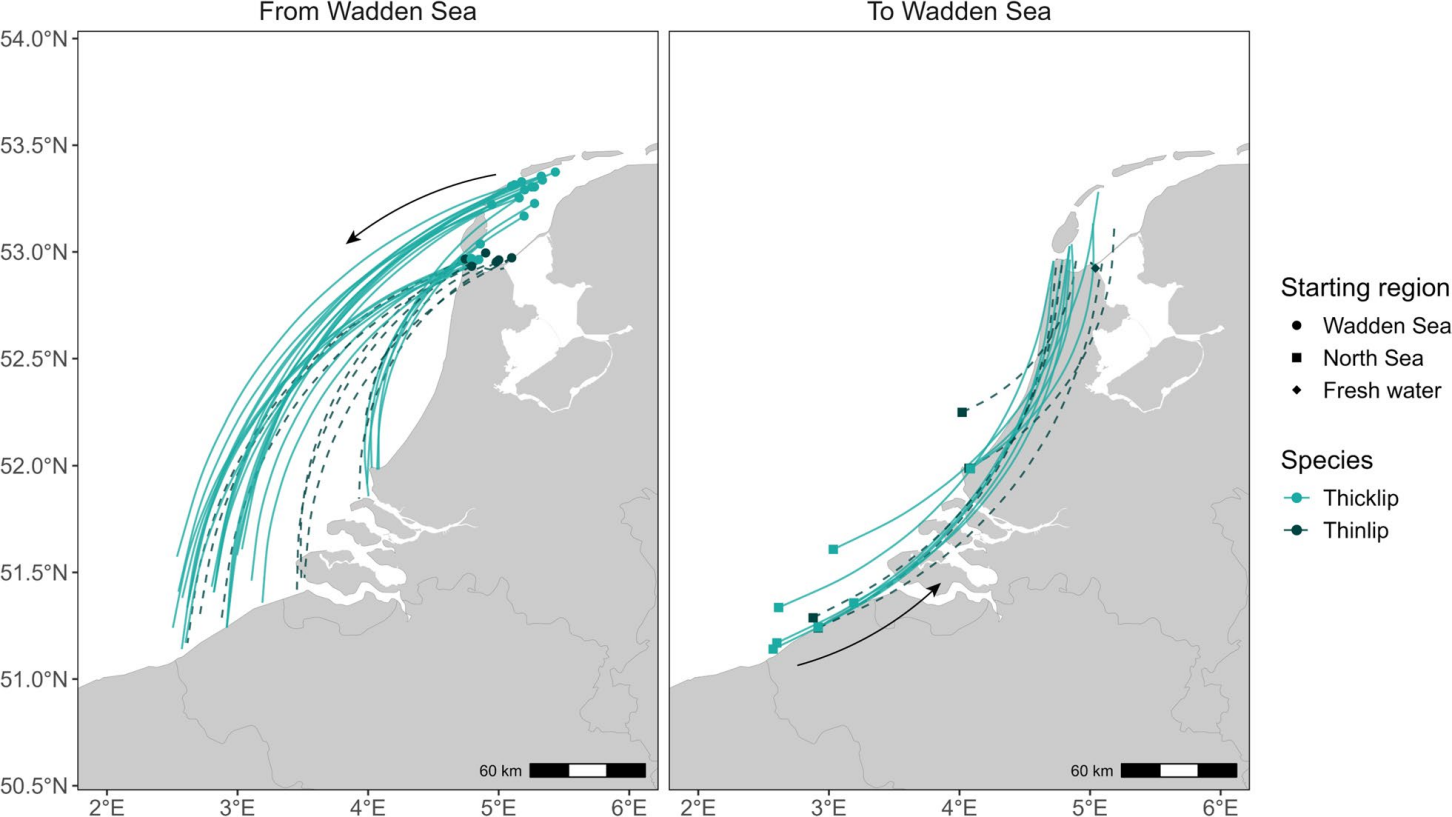
Regional connectivity of grey mullet movements between the Wadden Sea, North Sea, and fresh water (Lake IJsselmeer). Nodes represent the receiver stations where large-scale movements originated. Edges represent fish movements between regions and are right-hand curved from origin to destination

Movements between the Wadden Sea and North Sea were recorded in both directions for both species (Fig. 3). Movements from the Wadden Sea to the North Sea were recorded for 24 thicklip mullet and 8 thinlip mullet over 3 years (N = 34 recorded movements). Prior to departure, thicklip mullet were last detected in the northeast corner of the array and the Marsdiep Channel, while all thinlip mullet were last detected in the southwest of the Marsdiep. In the North Sea, most thicklip mullet were first redetected in Belgian waters (71%), with fewer detected along the Dutch coast (Haringvliet or Apelafico arrays). Thinlip mullet showed a slightly different pattern, with equal proportions moving to the Western Scheldt (ws1 array) as seen in Belgian waters (38% each). Return migrations were less frequent (9 thicklip, 5 thinlip), with most departures originating from Belgian waters for both species (N = 7 and N = 4, respectively) (Fig. 3).

Freshwater detections were limited to a single thinlip mullet that was detected by one receiver in the freshwater harbour of Den Oever in Lake IJsselmeer over two consecutive years (Fish ID: TNM08) (Fig. 3). This individual made multiple crossings between the Wadden Sea and fresh water, entering the lake in May of both years and returning to the Wadden Sea in May and June (Fig. S3, Supplementary Materials). Fish movements between the Wadden Sea and Lake IJsselmeer are constrained by the Afsluitdijk and require movement through sluice complexes located at Den Oever or Kornwerderzand (Fig. 1b).

### Patterns of occurrence in the Dutch Wadden Sea

**Fig. 4:**
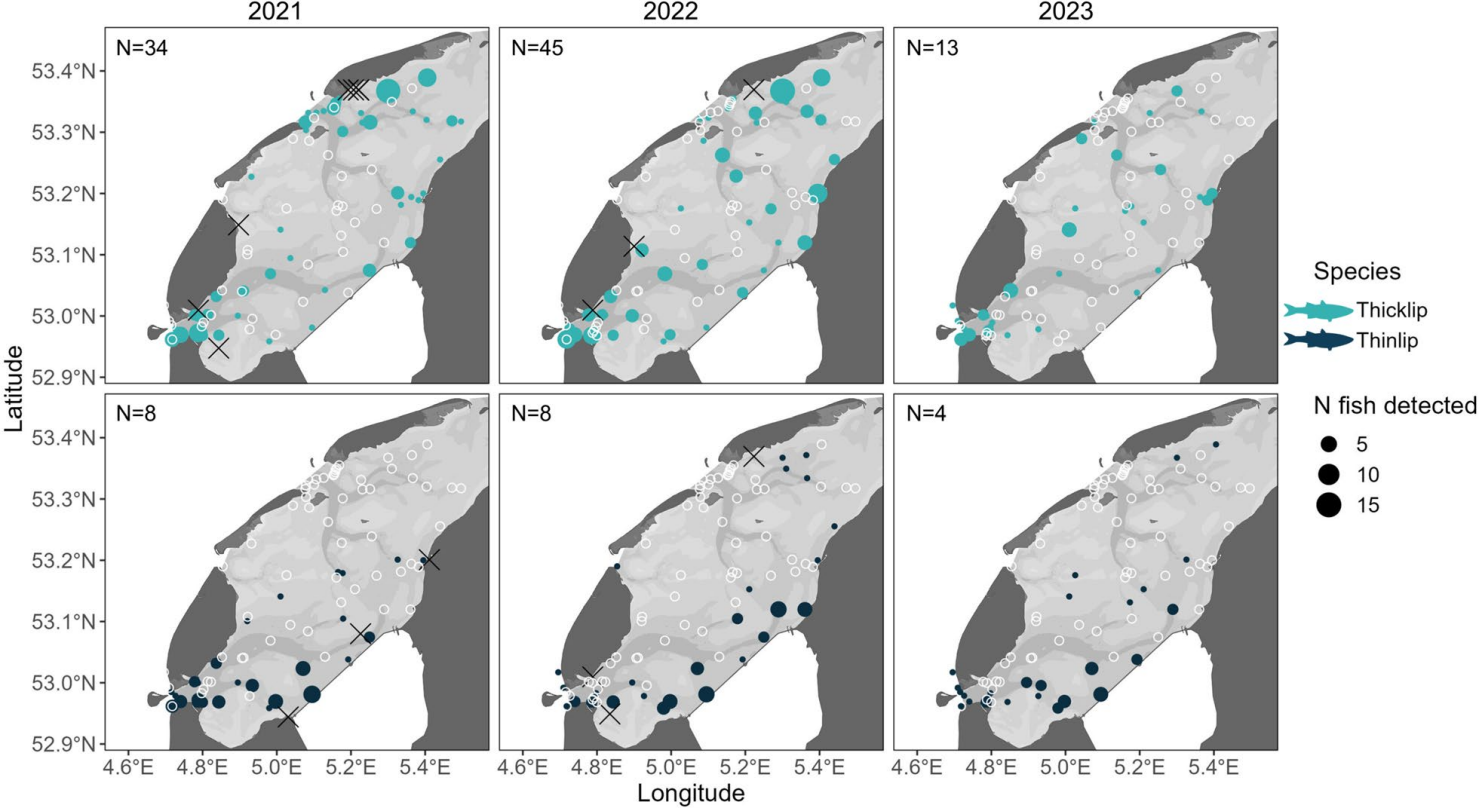
Distribution of acoustic detections for thicklip (N = 74) and thinlip mullet (N = 12) tagged in the western Dutch Wadden Sea and detected between May 2021 and October 2023. Open circles represent receiver stations without fish detections for the specified year. Fish were tagged in 2021 and 2022, with X’s marking the respective tagging locations for each year

Receiver stations in the Wadden Sea detected between 1 and 28 individuals over the full study period (median = 2.5, IQR = 1–6), with the highest overall numbers recorded near Terschelling in the northeastern corner of the array and in the Marsdiep Channel to the southwest (Fig. 1b, 4). In tagging years (*i.e.,* 2021 and 2022), peak fish counts were associated with, but not limited to stations close to tagging sites.

Thicklip mullet were detected across much of the array area over all years, regardless of whether tagging occurred (Fig. 4). Stations that did not detect thicklip mullet (considering only stations with adequate deployment periods, see Methods) were distributed throughout the array area but were often associated with the inner reaches of the main tidal gully systems (Fig. 4). Across all three years, persistent clusters of detection were located in the Marsdeip and Vlie basins (southwestern and northeastern gully systems, respectively) and near the port of Harlingen.

Thinlip mullet were mostly detected in the southwest of the array along the Aflsluitdijk and near a shallow area of intertidal flats known as the Balgzand (Fig. 4). Over the three years, thinlip mullet were detected by a median 27 stations per year and in only one of the three tidal inlets (Marsdiep) (Fig. 4). Sparse detections were recorded on receiver stations in the central and northern portions of the array, while many stations throughout the central, northern, and northeastern portions of the array did not detect any thinlip mullet throughout the three-year period.

**Fig. 5:**
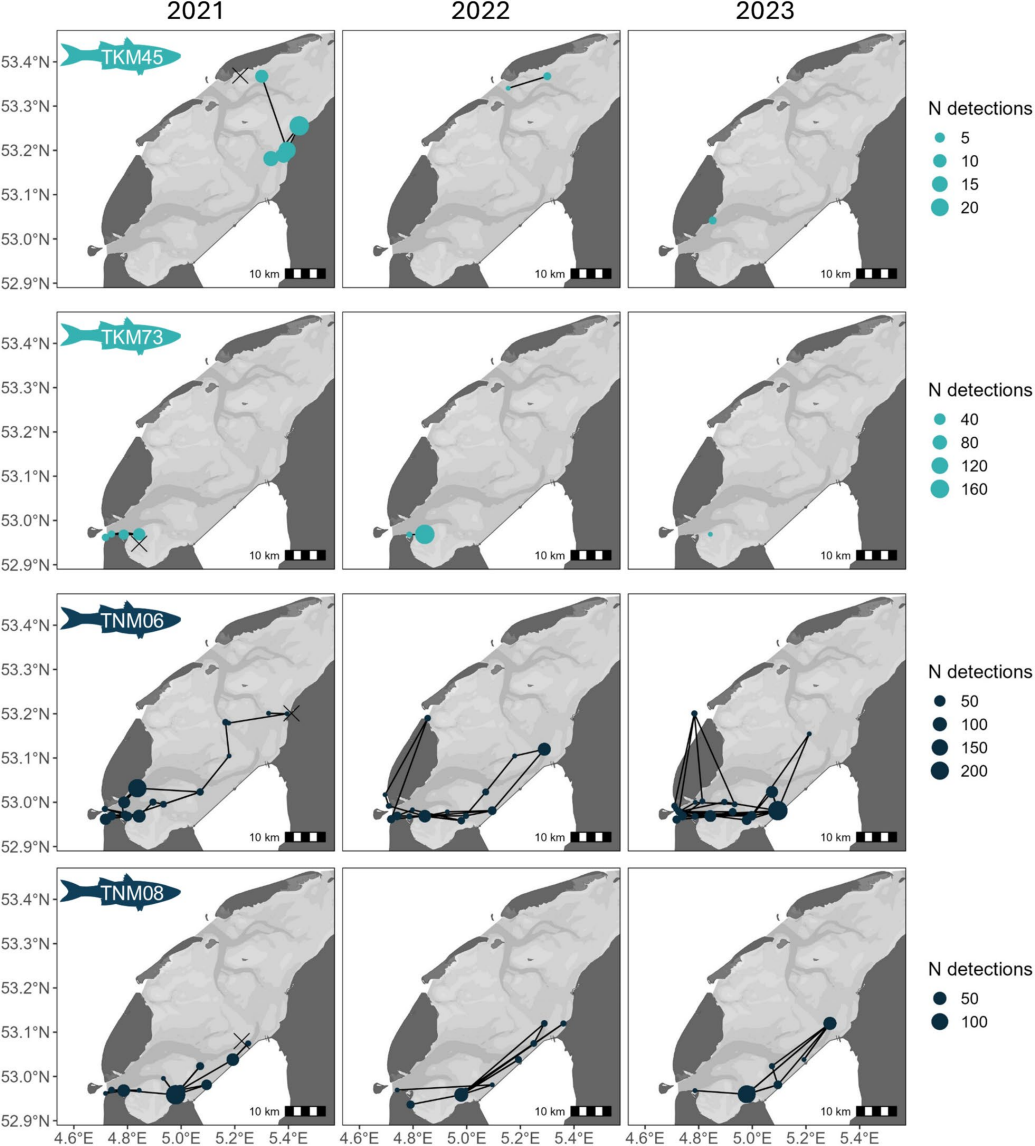
Examples of acoustic detection histories for two thicklip (Fish ID: TKM45, TKM73) and two thinlip (Fish ID: TNM06, TNM08) mullet tagged in 2021 and detected over three consecutive years in the western Dutch Wadden Sea. Tagging sites are denoted by an X

The local movements of tagged mullet varied widely among individuals (Fig. 5). While some individuals were detected by only a single receiver station (N = 19) or in a single year over the full study period (N = 61), others were detected for up to 3 consecutive years (N = 5) with a maximum of 19 unique stations in a single year (Fish ID: TNM06, 2021; Fig. 5). Thinlip mullet were the only individuals to be detected by receivers on the outer edges of the Wadden Sea (*i.e.,* North Sea shores of Wadden Islands), suggesting movements around the North Sea side of the Wadden Islands in lieu of transiting via the mudflats and gullies within the Wadden Sea (TNM06, Fig. 5). The detection histories of thicklip mullet typically had clusters of detections in discrete regions of the Wadden Sea, separated by vast areas without detected fish presence (*e.g.,* TKM45, Fig. 5). This is contrasted by many of the individual detection histories of thinlip mullet, where detections appeared more contiguous.

### Timing and duration of Wadden Sea residence

**Fig. 6:**
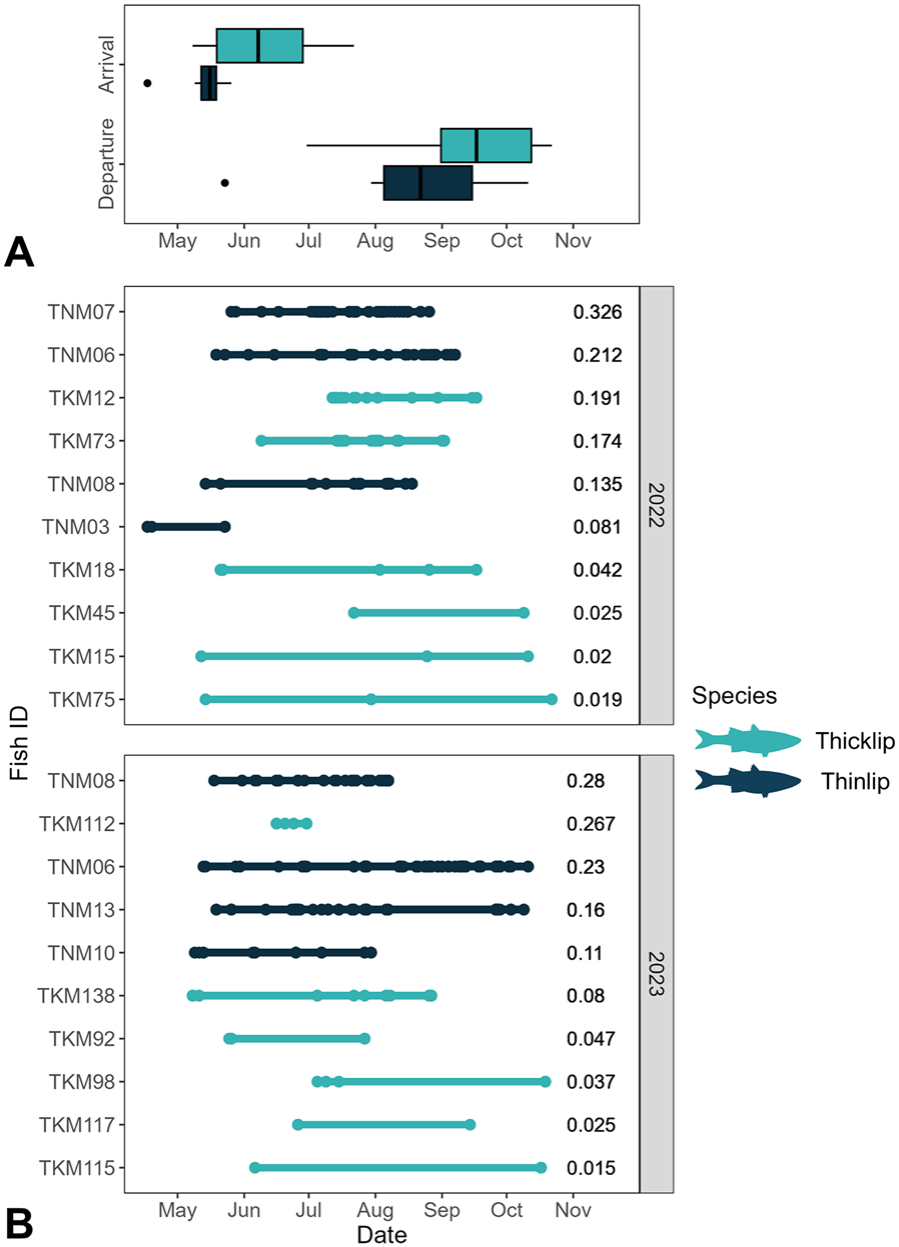
Seasonal residence of individual thicklip mullet (N = 12) and thinlip mullet (N = 6) detected in the western Dutch Wadden Sea in subsequent years following acoustic tagging. **A** Timing of arrival and departure as inferred from first and last detections. **B** Timing of individual detections (dots) and residence duration (shaded bars). Local residency index (RI) values are displayed to the right of each detection profile (RI = N detection days/residence duration). Detection profiles are arranged in order of descending RI values

For both grey mullet species, fish were first detected in the Wadden Sea primarily in spring, with last detections spread over a broader period from spring to autumn (Fig. 6a,c, Table 3). Thicklip mullet returned to the Wadden Sea from early May to early July (median = June 7th), while departure dates spanned from the end of June to late October (median = Sept 17th). Thinlip mullet arrived from mid-April to late May (median = May 16th), with departures from late May until mid-October (median = August 22nd). Arrival dates differed significantly between the two species (χ^2^ = 4.84, df = 1, *p* < 0.05), meanwhile no difference in departure timing was detected (χ^2^ = 2.39, df = 1, *p* = 0.12; Fig. 6a). In addition, there was no difference in the duration of Wadden Sea residence exhibited by returning thicklip and thinlip mullet (χ^2^ = 0.05, df = 1, *p* > 0.05; Fig. 6c, Table 3). In contrast, RI differed significantly between the two species with higher values for thinlip mullet (χ^2^ = 6.88, df = 1, *p* < 0.05; Fig. 6b, Table 3).

**Table 3:** Arrival and departure dates, residence duration, and Residency Index (RI) for thicklip mullet (N = 12) and thinlip mullet (N = 6) tagged in the western Dutch Wadden Sea

Species	Category	Median (DOY)	Median date	IQR (DOY)
Thicklip	Arrival	158	June 7	138–178
	Departure	259	Sept 17	243–285
	Residence duration	97		77–124
	RI	0.040		0.023–0.104
Thinlip	Arrival	135	May 16	131–138
	Departure	233	Aug 22	216–257
	Residence duration	94		82–121
	RI	0.186		0.129–0.243

Data include only detections recorded in return years and individuals detected for a total duration of > 5 days. Median values and interquartile ranges (IQR) are shown as day-of-year (DOY)

### Space use in the Dutch Wadden Sea

**Fig. 7:**
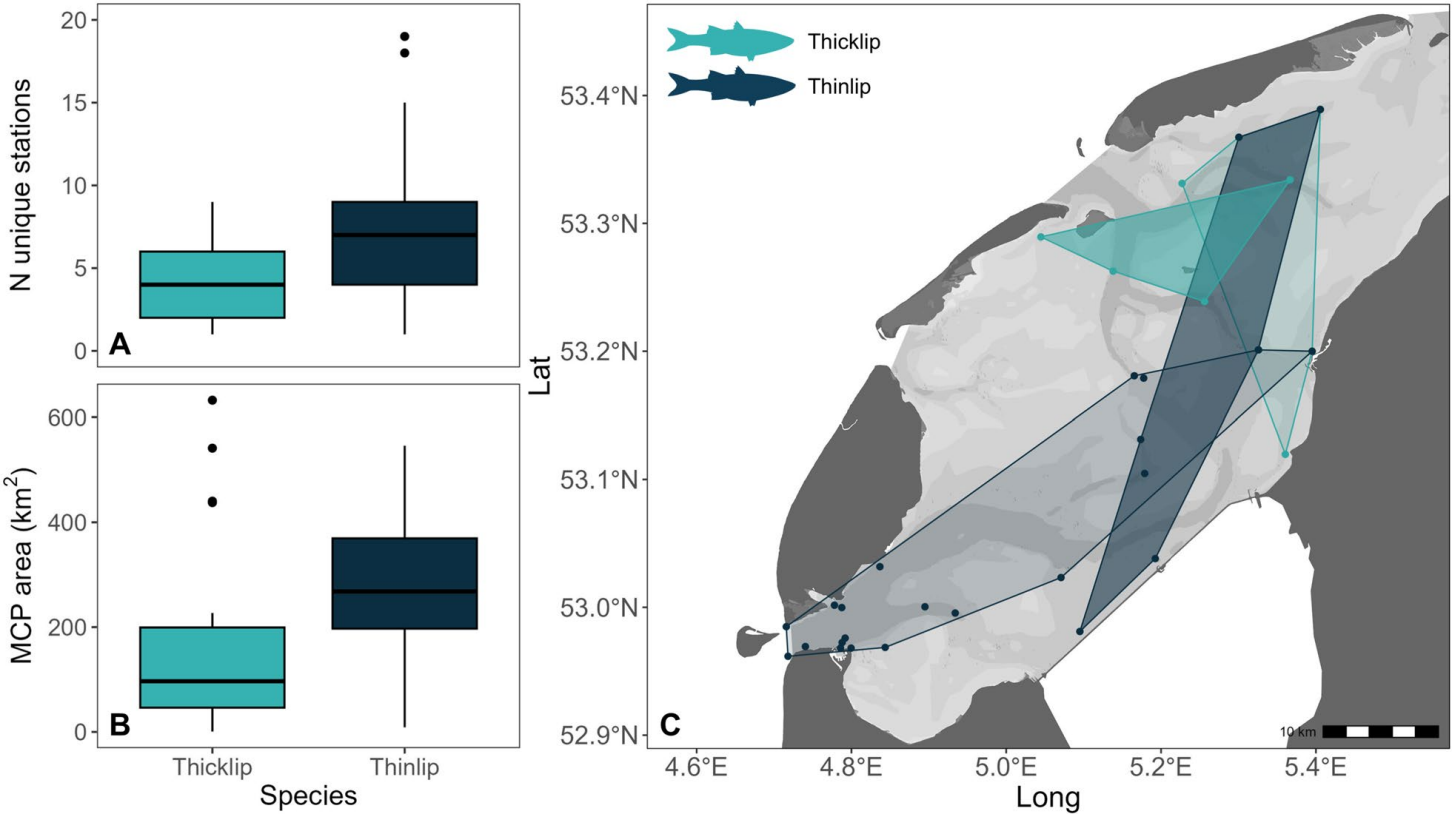
Annual space use of acoustic-tagged thicklip (N = 36) and thinlip mullet (N = 9) during the summer residence period in the western Dutch Wadden Sea. The number of unique stations (**A**) and area of minimum convex polygons (MCP area) (**B**) were calculated per individual and year, and aggregated by species. Panel **C** illustrates example individual MCP areas representing the median (darker polygons) and upper (lighter polygons) quantile MCP areas for each species. Included individuals were detected in the Wadden Sea for > 5 days at ≥ 3 receiver stations per year

Space use differed between species across all three metrics. First, thinlip mullet were detected by significantly more unique receiver stations per year than thicklip mullet (χ^2^ = 8.46, df = 1, *p* < 0.05; Fig. 7a). Per individual, the median number of stations per year was 7 for thinlip mullet (IQR = 4–9) and 4 for thicklip mullet (IQR = 2–6). The number of receiver stations per individual did not vary significantly between tracking years for either species (thicklip: χ^2^ = 2.61, df = 1, *p* = 0.11; thinlip: χ^2^ = 1.29, df = 2, *p* = 0.52).

This pattern was consistent with minimum convex polygon (MCP) area, with thinlip occupying significantly larger areas than thicklip mullet (χ^2^ = 9.37, df = 1, *p* < 0.05; Fig. 7b). The median MCP area for thinlip mullet was 268 km^2^ (IQR = 197–369), compared to 97 km^2^ (IQR = 46–199) for thicklip mullet (Fig. 7b,c). No significant differences in MCP area were observed between tracking years for either species (thicklip: χ^2^ = 0.05, df = 1, *p* = 0.83; thinlip: χ^2^ = 0.72, df = 2, *p* = 0.70). These data suggest that thinlip mullet exhibit wider-ranging movements or greater use of gully systems compared to thicklip mullet. Conversely, thicklip mullet may utilise smaller areas or prefer shallow areas and intertidal flats with lower detection probability.

The local residency index, RI_*stat*_, revealed differences in the frequency of recurrence at unique receiver stations and the distribution of fish presence for the two grey mullet species. RI_*stat*_ differed significantly between species, with higher values for thicklip (median = 0.18, IQR = 0.10–0.34) than for thinlip (median = 0.05, IQR = 0.02–0.10) (χ^2^ = 51.88, df = 1, *p* = < 0.05).

**Fig. 8:**
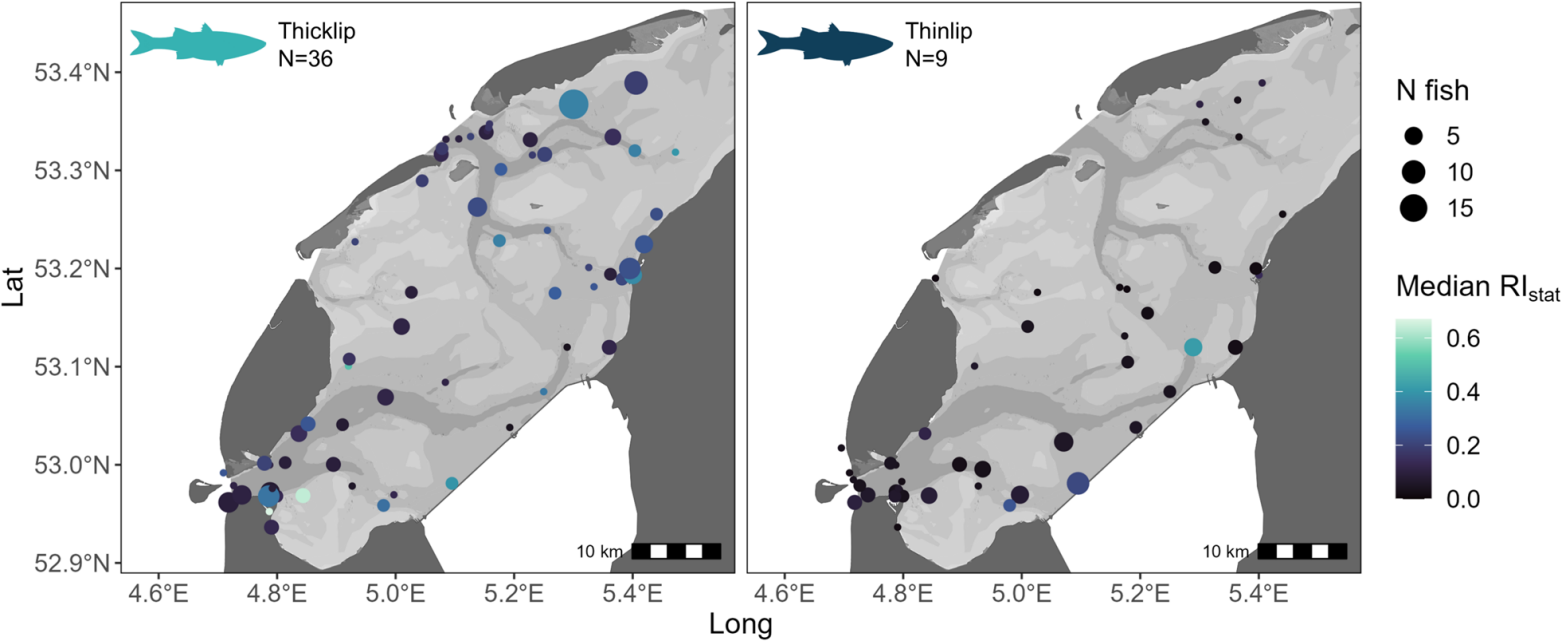
Median local residency index (RI_*stat*_) per acoustic receiver station in the western Dutch Wadden Sea. Data include detections from 2021–2023 and are grouped by detected species: thicklip mullet (left panel) and thinlip mullet (right panel). Only individuals detected for a period of > 5 days in a given year are included

Within each species, median RI_*stat*_ was calculated for each receiver station, revealing differences in the distribution of fish presence throughout the array. For thicklip mullet, stations with the highest median RI_*stat*_ were concentrated near three major harbours, located in Den Helder in the southwest, on the south shore of Texel, and to the east in Harlingen (Fig. 8). For thinlip mullet, three receiver stations located near the southwestern and northeastern ends of the Aflsuitdijk had the highest median RI_*stat*_, followed by stations located outside the harbours of Harlingen and Oudeschild (Texel), at the northeastern edge of the array, and in the Marsdiep Channel (Fig. 8).

## Discussion

This study used acoustic telemetry to reveal local and broad-scale movement patterns of adult grey mullets in the temperate waters of the Wadden Sea and southern North Sea. Using an extensive receiver network in the western Dutch Wadden Sea—an important coastal feeding ground where no prior fish tracking studies have been conducted—we compared the migration timing and space use of two sympatric grey mullet species for the first time in this region. Detection data from receiver arrays spanning over 500 km along the Dutch and Belgian coasts (https://www.lifewatch.be/etn/) provided insights into broad-scale migrations, with repeated visits to the Wadden Sea, North Sea, and fresh water. Tagged fish were detected in the North Sea over ten months of the year, while presence in the Wadden Sea and Lake IJsselmeer was limited to the warmer period from spring to autumn, reinforcing their classification as seasonal visitors [36, 66]. Within the Wadden Sea, thinlip mullet arrived earlier in spring, were detected more frequently during the summer residence period, and occupied larger areas than thicklip mullet, highlighting differences in movement ecology. Thinlip mullet also exhibited a stronger association with freshwater outflows along the Afsluitdijk, reflecting their documented affinity for fresh water. These findings provide valuable insights into the timing and locations of seasonal occurrence, informing species-specific management.

**Fig. 9:**
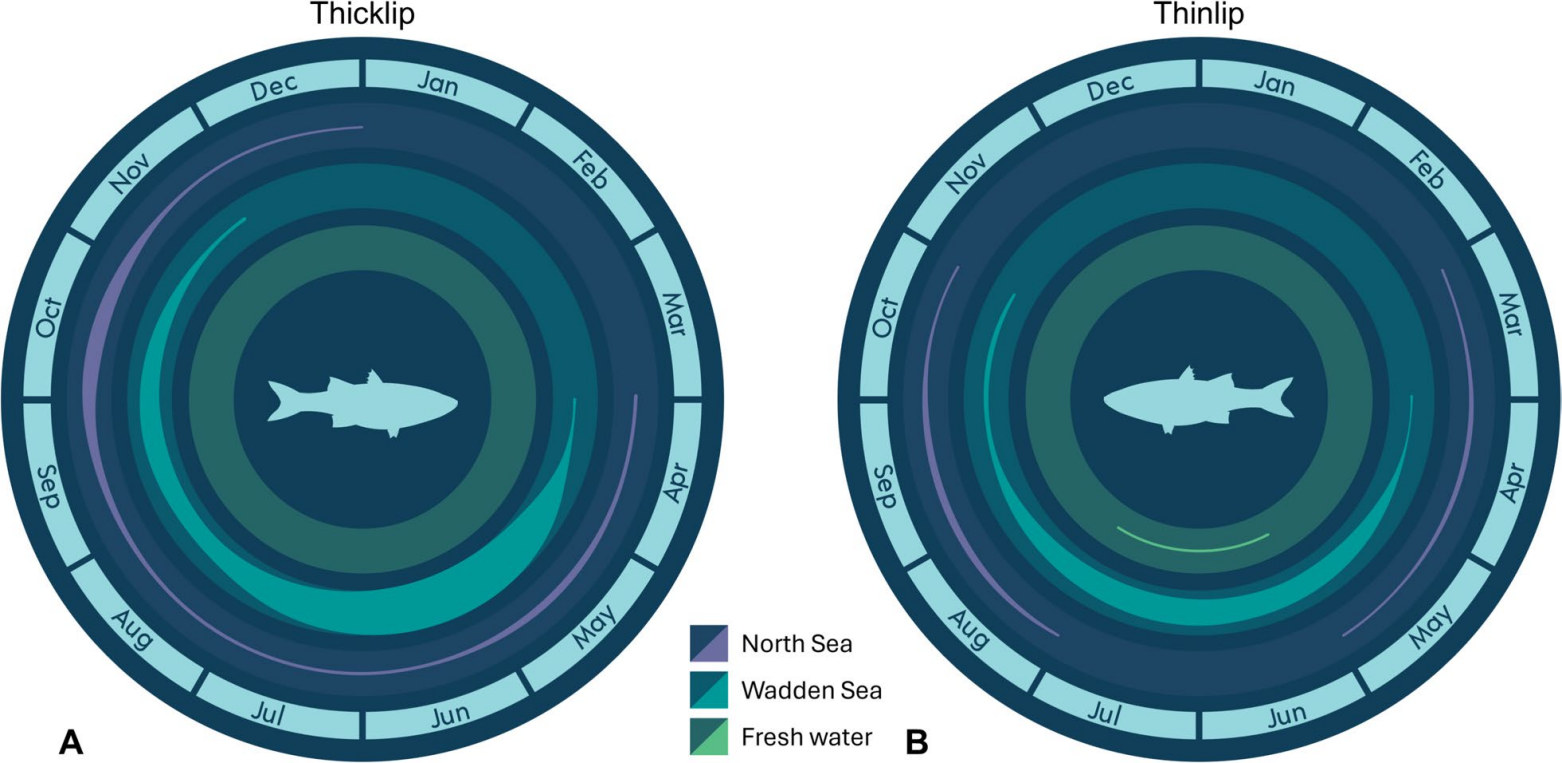
Relative monthly presence of acoustic-tagged thicklip (**A**) and thinlip (**B**) mullets across three aquatic regions. Coloured bands represent the timing and region of fish presence, with band width approximating the relative number of individuals detected in each region and month

Tagged individuals from both species demonstrated similarities in large-scale space use, migration routes, and winter habitats in the southern North Sea. The observed pattern—leaving the Wadden Sea for the North Sea in autumn and returning in spring (Fig. 9)—aligns with the behavior of other migratory Mugilids, which favor coastal and estuarine habitats in warmer months and migrate offshore in autumn in preparation for spawning [78, 79]. In the North Sea, particularly near the Rhine-Meuse river mouth (Haringvliet array), grey mullets were detected not only during migration, but also throughout the summer, overlapping with their summer residence period in the Wadden Sea. This seasonal overlap, coupled with similar residence durations recorded in both regions (Figure S2, Supplementary Materials), suggests that mullets tagged in the Wadden Sea may use this area as an alternative summer foraging ground.

While mullet presence was detected across all seasons, a lack of detections in January and February suggests that tagged fish migrated to locations beyond the reach of the included receiver networks (Fig. 9). This absence of detections coincides with the presumed spawning period for thicklip mullet in European waters (January–April) [40]. To date, the only known spawning ground for this species in the northeast Atlantic is off southwestern England near the Isles of Scilly, more than 800 km from the Wadden Sea [40]. However, no tagged fish were detected by receiver arrays on the UK side of the English Channel, despite extensive receiver coverage and equipment compatibility. In contrast, limited data reporting or incompatibility in telemetry technologies may have impeded the detection of our tagged fish in more eastern regions [60], despite reports that the distribution of thicklip mullet extends into German and Danish waters [66]. While our data cannot confirm whether fish tagged in the Wadden Sea migrate to the known spawning grounds or other habitats beyond receiver coverage, the use of data storage tags (*i.e.,* bio-loggers), capable of recording high-resolution temperature and depth profiles, offers a promising method to identify aggregation sites and potential spawning grounds beyond the reach of existing receiver networks [36].

European grey mullets exhibit notable differences in habitat use, particularly in relation to their occurrence in fresh water. Thinlip mullet possess the highest osmoregulatory capacity among North Atlantic mullet species, enabling them to undertake extensive migrations between freshwater and coastal habitats [2, 3, 43]. Reports from various regions, including Portugal, France, and Morocco, describe migrations of thinlip mullet extending hundreds of kilometres upstream, followed by prolonged periods of fresh water residency [3, 6, 51, 52, 55, 64, 65]. In Portugal’s Tagus river system, thinlip mullet also display partial migration, with some individuals residing year-round in fresh water [51]. Among our tagged fish, only one thinlip mullet was detected in fresh water. However, the distribution of detections across the Wadden Sea array (as indicated by RI_*stat*_) suggests that thinlip mullet are more drawn to areas of reduced salinity than thicklip mullet. Thinlip mullet were primarily detected along the southern edge of the Wadden Sea, particularly near the Afsluitdijk’s sluice complexes at Den Oever and Kornwerderzand, where freshwater discharges into the Wadden Sea and mixes with the tidal saline waters [70]. In contrast, thicklip mullet were more evenly distributed across the Wadden Sea, including frequent occurrence near tidal inlets where salinity is closer to that of the North Sea [70]. While the small sample size for thinlip mullet may explain the lack of additional freshwater connectivity, the controlled waterways of the Netherlands likely also hindered upstream migration in our study area.

In our study, repeated movements of a sole thinlip mullet between the Wadden Sea and Lake IJsselmeer likely occurred via the Den Oever sluice complex of the Afsluitdijk, which operates under a Fish Friendly Management Regime to facilitate fish migration [39]. In the Netherlands, grey mullets are frequently observed in large numbers in freshwater canals, however, connections between fresh and coastal waterways are almost entirely controlled by dams, pumping stations, and sluices, which restrict fish passage [39, 76, 83, 84]. Fragmentation caused by artificial barriers likely limits thinlip mullets migration and, in cases where partial migration occurs, may contribute to separation between upstream and coastal contingents. Research in other fragmented river systems have revealed the importance of freshwater access for thinlip mullet [46, 52, 55]. Expanding tagging efforts to include more individuals from both freshwater and marine habitats could provide valuable insights into population connectivity and the efficacy of fish-friendly sluice management strategies. Such efforts, alongside continued monitoring of artificial fish passages and sluice gates using technologies like PIT tags (passive integrated transponders) or acoustic telemetry (*e.g.,* RBVV2 array) would clarify the role of freshwater habitats for thinlip mullets. This monitoring could also enhance knowledge of fishway efficacy, particularly at sites like the Afsluitdijk, where new fishways are currently under construction.

In the Dutch Wadden Sea, both thicklip and thinlip mullet were seasonally present between April and October, aligning with peak water temperatures and primary productivity in this shallow coastal environment [18]. The migration timing of thinlip mullet differed slightly between the Wadden Sea and other European habitats. In Spain and Portugal, thinlip mullet begin their upstream migrations in March/April, reaching a peak in July/August [1, 3, 52, 55, 65], and return downstream from autumn through early winter (Nov–Feb) [2, 40], much later than observed in our study. For thicklip mullet, limited data suggest peak spawning occurs from January to April [40], potentially aligning with our observed migration timing. However, tagged thicklip mullet arrived earlier and departed later than those caught in the historical NIOZ fyke sampling survey, conducted since the 1960s in the Wadden Sea’s Marsdiep Channel [11, 77]. Differences in timing could be due to incomplete receiver coverage and low detection efficiency in the Wadden Sea’s tidal inlets [35], which may have resulted in missed detections for tagged fish entering and exiting the system.

The observed disparities in station count and area size between thicklip and thinlip mullet likely reflect ecological differences in their space use and movement patterns within the Wadden Sea. Thinlip mullet may exhibit greater mobility and a broader spatial range, resulting in more frequent detections across a larger number of receivers. Alternatively, the distribution of receiver stations—primarily in deeper tidal inlets and gullies—may have influenced the observed detection patterns. It was necessary to deploy receivers in deeper areas to avoid exposure at low tide, but this may have biased detections toward fish frequenting these habitats, potentially overlooking individuals with a preference for shallow coastal zones and mudflats outside of detection range. If our results are indeed a reflection of deployment location, this could point to behavioural differences between the two species, with thinlip mullet spending more time in deeper waters and thicklip mullet preferring to use shallow areas where they cannot be detected. Individual trajectories of thicklip mullet also support this hypothesis, showing broad spacing between clusters of detections, which could indicate undetected movements through shallow areas while traversing the Wadden Sea.

Classical niche theory provides a framework for understanding the behavioural differences observed between these closely related species. Niche partitioning, for example through dietary or spatial segregation, reduces competition and facilitates the stable coexistence of closely related species [23, 24, 47, 49]. Dietary segregation in particular, is considered one of the most important factors promoting diversity and defining the structure of fish communities [13, 28, 38, 63]. For example, in coral reef systems, complementary feeding behaviours allow complex algal-dependent fish communities to exist [8, 16, 17, 37]. In grey mullets, sympatry relates to both dietary specialisation (based on preferred sediment grain sizes and feeding times) as well as high prey abundance, which prevents competitive exclusion even in cases of dietary overlap [10, 20].

Differences in grazing habitat and preferred grain sizes have yet to be established for thicklip and thinlip mullet in the Wadden Sea. However, as seen in African and Mediterranean estuaries [10, 20], the high summer productivity in the Wadden Sea may allow redundancy in local feeding behaviours. Furthermore, stable isotope analysis has revealed slight variations in diet among the grey mullet species occupying the Dutch Wadden Sea, hinting towards potential dietary segregation. Thicklip mullet were found to occupy a higher trophic level (2.3) than golden (2.1) and thinlip mullet (2.0) [56]. These dietary differences, along with variations in osmoregulatory capacity, could explain the observed differences in spatial distribution for thicklip and thinlip mullet and indicate niche separation. The Wadden Sea encompasses a variety of intertidal and subtidal habitats, including mussel and oyster reefs, seagrass beds, and sandy or muddy substrates with varying grain sizes [7, 26]. Future studies seeking to unravel potential differences in habitat use and dietary segregation should consider incorporating such features of spatial heterogeneity.

## Conclusions

This study represents the first detailed comparison of the movement ecology of thicklip and thinlip mullet in temperate European waters. These findings enhance our understanding of the migratory behaviours of these two sympatric species, refining the timing and duration of seasonal presence across coastal, offshore, and freshwater habitats. Notably, these results provide the first evidence of recurrent movements between the Wadden Sea and adjacent regions. Within the Wadden Sea, we uncovered differences in the seasonal timing of arrival, detection frequency, and spatial extent of space use between the two species.

This study faced limitations resulting from the design of the SWIMWAY acoustic array, which provides incomplete coverage of the study area due to challenging environmental conditions and complex bathymetry. Missed detections in the tidal inlets may have influenced estimates of arrival and departure times. Meanwhile, the placement of receiver stations in subtidal waters might have biased detections toward individuals favoring the gully systems over tidal flats. Future research into the space use of tagged fish in the Wadden Sea should employ analytical methods that can account for unequal detection probabilities due to receiver placement.

To further explore the processes underlying these behavioural differences, future studies should investigate spatial variation in environmental factors such as salinity and sediment grain size. This could reveal whether niche partitioning through dietary segregation or physiological adaptations supports the coexistence of these grey mullet species.

Overall, our findings offer valuable insights for future study design and could guide management strategies aimed at enhancing the resilience of European grey mullets in the Dutch Wadden Sea.

## Supplementary Information

Additional file 1.

Additional file 2.

## Data Availability

The datasets generated by the authors during the current study are archived and published in the NIOZ Digital Archiving System. Additional detection data were sourced from the database of the European Tracking Network data portal: (https://www.lifewatch.be/etn/), developed by the Flanders Marine Institute as part of the Flemish contribution to LifeWatch.
