# Supplementary material for: Seasonal coastal residency and large-scale migration of two grey mullet species in temperate European waters
Source: Mov Ecol. 2025 Jan 11;13:2. doi: 10.1186/s40462-024-00528-z (PMC11724599; doi:10.1186/s40462-024-00528-z)
Supplement: Supplementary file 2 — Additional file 2. [file 40462_2024_528_MOESM2_ESM.docx]

**Supplementary Materials**

**Seasonal coastal residency and large-scale migration of two grey mullet species in temperate European waters**

Edwards, J.E., Buijse, A.D., Winter, H.V., Bijleveld, A.I.

1. ***Fish tagging – tag selection and fish size***

Using the 2% body weight rule as a general guideline (Jepsen et al., 2005), the minimum fish size appropriate to for each tag type was estimated. Acoustic transmitters included the following models and specifications: V13-1x (135-235 s nominal delay, power output 152 db [re 1 µPa at 1m], 915 d battery life), V13AP-1x (50-100 s nominal delay, power output 152 db, 278-293 d battery life), and V16-4x (40-80 s nominal delay, power output 158 db, 1415 d battery life) (Innovasea Systems Inc., Bedford, Canada). For some individuals, a data storage tag with float collar (G5 Long-Life DST; Cefas Technology Ltd., Suffolk, UK) was implanted alongside the acoustic transmitter (used with V13-1x transmitters only). Assuming the weight of the smallest acoustic transmitter used in this study (V13-1x, 11 g in air), the minimum fish weight was approximated as 550 g. This corresponded to a total length of 41.5 cm as per the length-weight relationship for thicklip grey mullet as stated on FishBase (Froese et al., 2014; Froese & Pauly, 2023). For larger V16 transmitters, minimum fish size was 650 g, corresponding to approximately 50 cm total length. For double tagging, in which both a V13 acoustic transmitter and a G5 DST were internally implanted, individuals were required to have a minimum weight of 1600 g and a length of 49.7 cm (for details see **Table S2**, Supplementary Materials). Acceleration data from V13AP tags and archived data from G5 DSTs were not analysed as these data were beyond the scope of the current study.

1. ***Assessing post-tagging mortality***

During the study period, eight cases of potential fish mortality were identified (**Fig S1**). Post-tagging mortality was determined based either on the recapture of acoustic or data storage tags or via examination of individual detection profiles. For six thicklip mullet (5.6% of tagged population) and one golden grey mullet (30% of tagged population), mortality was determined via the return of internal acoustic transmitters and/or data storage tags by fisherman and beach combers. The remaining two thicklip mullet were presumed dead based on an extended sequence of repeated detections at a single station (TKM19, TKM113; **Fig. S1**). This pattern is typical of dead fish or expelled transmitters resting on the sea bed near a receiver (Klinard & Matley, 2020) and is unlikely to represent sedentary behaviour in this highly mobile species.

1. ***Residency in the southern North Sea***

Due to the limited number of detection days per tagged fish, residence duration in the North Sea could only be assessed in two of the five subregions, namely Belgian inshore waters and the Haringvliet estuary (**Fig. S2**, Supplementary Materials). In Belgian inshore waters, one thicklip mullet stayed for 9 days, while two thinlip mullet resided for 7 and 24 days (median = 15.5). Additionally, four thicklip and one thinlip mullet were detected for periods of 1-5 days. In the Haringvliet estuary, five thicklip mullet had a median residency of 62 days (range = 20–198), and two thinlip mullet had a residency of 6 and 32 days. Three additional thicklip mullet were detected, each for a single day. At all other locations, individuals were detected for no more than one or two days.

1. ***Thinlip detections in Lake IJsselmeer***

TNM08 was first detected in fresh water in its second tracking year between May 28 and June 6, 2022 (6 detection days) before the returning to the Wadden Sea from July 1 to Aug 17, 2022 (6 detection days) and subsequently departing to the North Sea where it was detected for the second consecutive year by a single receiver in the Belgian ‘bpns’ array (Aug 29, 2022). In its third tracking year, TNM08 was first detected in the Wadden Sea from May 17-30, 2023 (2 detection days) before re-entering lake IJsselmeer where it remained from May 22-28, 2023 (7 detection days) prior to returning to the Wadden Sea on May 30^th^ (**Fig. S3**). It was last detected in the Wadden Sea for a further 21 detection days in June, July, and August before its final detection in the south west of the array on Aug 6, 2023.

**
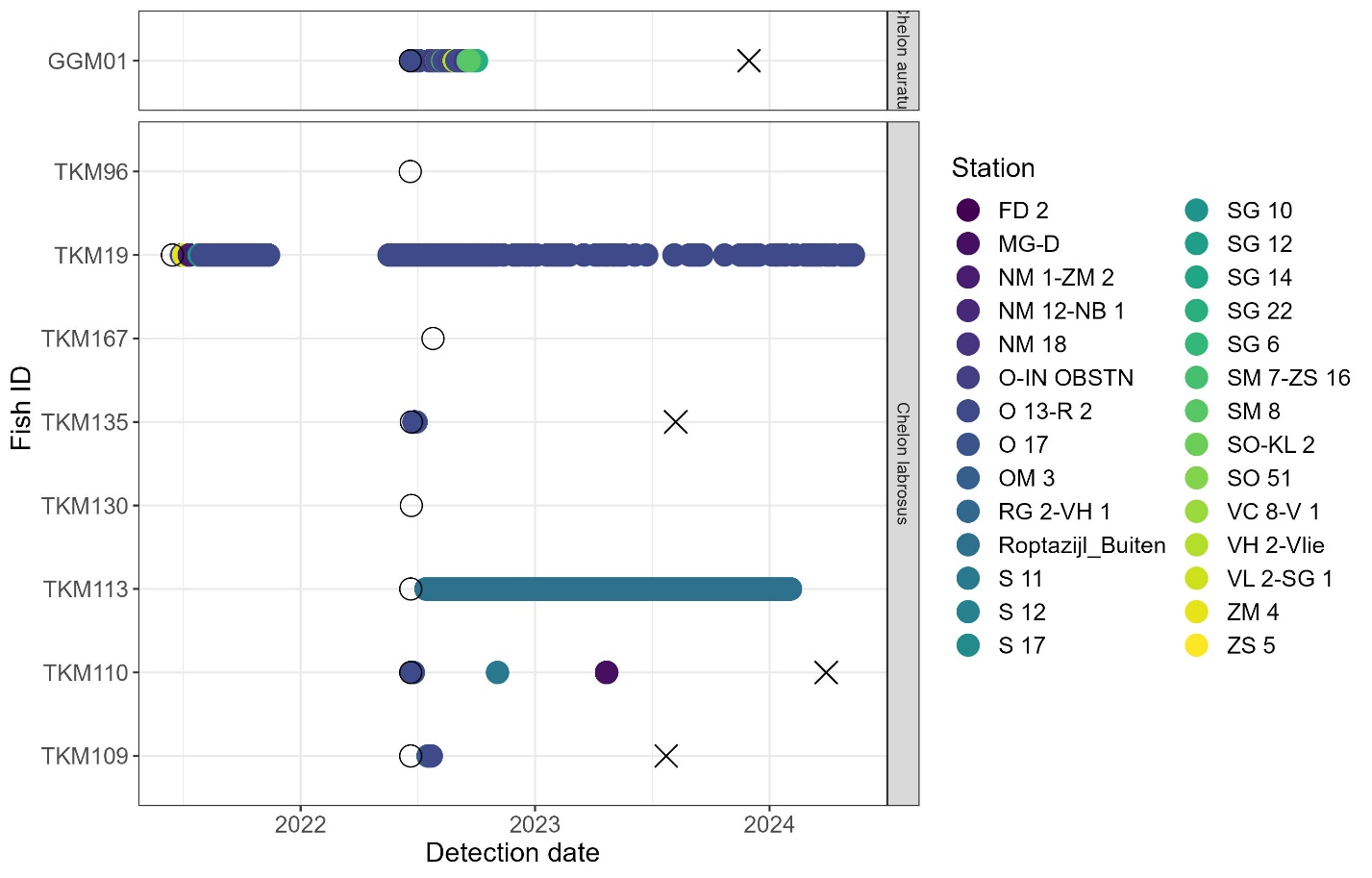
**

**Figure S1 |** Detection profiles for individual golden grey mullet (*C. auratus*) and thicklip grey mullet (*C. labrosus*) tagged with acoustic and archival telemetry tags in the western Dutch Wadden Sea and classified as mortalities. Open circles indicate the date of tagging and release for each fish, and ‘X’ denotes the date of data storage tag recovery used to confirm the deaths of some double-tagged individuals.


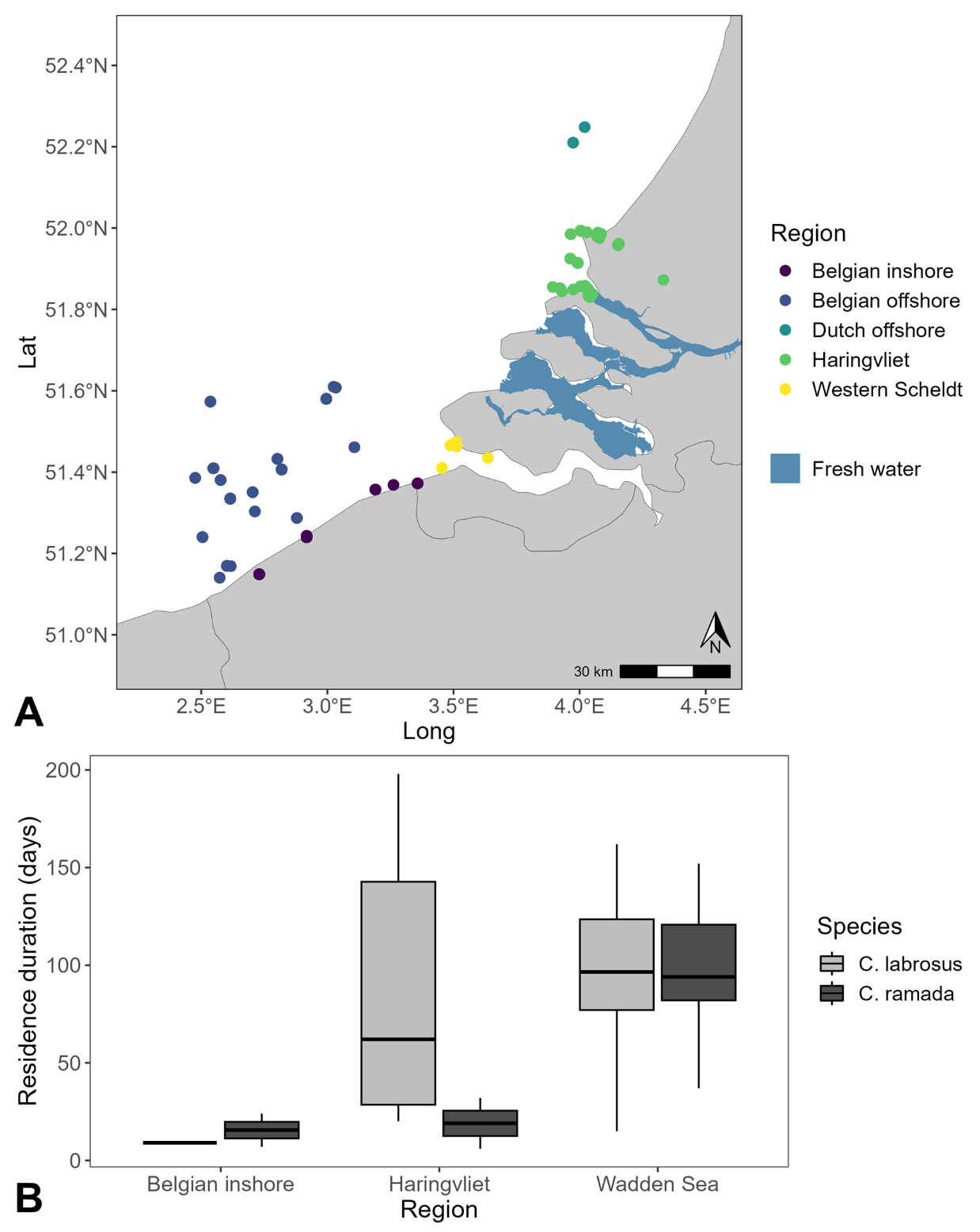


**Figure S2 |** Locations and duration of temporary residency of acoustic-tagged grey mullets (*Chelon labrosus*, *Chelon ramada*) in the southern North Sea and Dutch Wadden Sea. A) Positions of acoustic receiver stations in the southern North Sea with detections of tagged grey mullet. B) Residence duration of grey mullets detected for >5 days a year in two regions of the southern North Sea (Belgian inshore waters and the Haringvliet estuary) and the Dutch Wadden Sea.


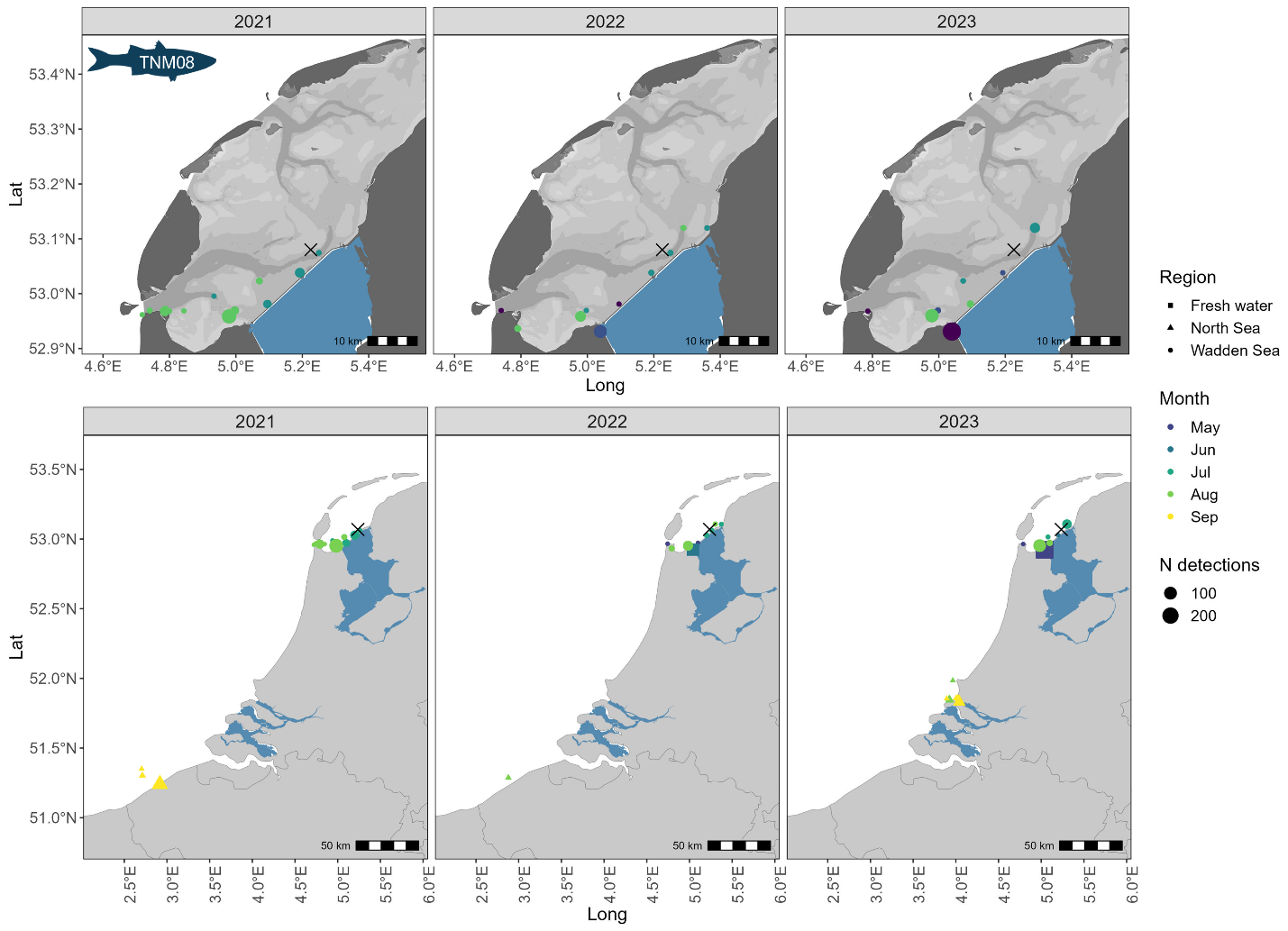


**Fig S3 |** Acoustic detections of a thinlip mullet (Fish ID: TNM08) in the southern North Sea, the western Dutch Wadden Sea, and Lake IJsselmeer between Aug 2021 and May 2023. Blue shaded areas indicate fresh water. X indicates the location of tagging on July 1, 2021. Excluding detections from receivers in the SWIMWAY array, data were obtained via the database of the European Tracking Network: https://www.lifewatch.be/etn/.

**Table S1 |** Specifications for acoustic transmitters implanted in grey mullets (*Chelon spp.*) in the western Dutch Wadden Sea in 2021 and 2022.

| **Tag family** | **Est tag life (days)** | **Power (L/H)** | **Min Delay (sec)** | **Max Delay (sec)** | **Sensor type** | **Range** | **Units** | **Accelerometer Samples (/sec)** | **N tags deployed** |
| --- | --- | --- | --- | --- | --- | --- | --- | --- | --- |
| V13AP-1x-BLU-1 | 278 | H | 50 | 100 | A | +/- 4.9 | m/s² | 12.5 | 10 |
| V13AP-1x-BLU-1 | 278 | H | 50 | 100 | P | 34 | Meters |  |  |
| V13AP-1x-BLU-1 | 293 | H | 50 | 100 | A | +/- 4.9 | m/s² | 12.5 | 10 |
| V13AP-1x-BLU-1 | 293 | H | 50 | 100 | P | 34 | Meters |  |  |
| V13-1x-BLU-1 | 915 | H | 135 | 235 |  |  |  |  | 94 |
| V16-4x-BLU-1 | 1415 | H | 40 | 80 |  |  |  |  | 12 |

**Table S2 |** Estimated appropriate body sizes of tagged grey mullets (*Chelon spp.*) based on tagging regime and the 2% body weight rule and the length-weight relationship of the thicklip grey mullet (Froese & Pauly, 2023).

| **Species** | **Length at maturity (mm)** | **Tag model** | **Tag weight in air (g)** | **Min fish weight in air (g)** | **Min fish length (cm)** | **Age-class** |
| --- | --- | --- | --- | --- | --- | --- |
| *C. labrosus* | 295 | V13-1H | 11 | 550 | 41.5 | Adult |
|  |  | V13AP-1H | 13 | 650 | 43.7 | Adult |
|  |  | V16-4H | 24 | 1,200 | 49.3 | Adult |

**Table S3 |** Presumed mortalities of golden grey mullet (*C. auratus*) and thicklip grey mullet (*C. labrosus*) tagged with acoustic and archival tags in the western Dutch Wadden Sea.

| **Species** | **Fish_ID** | **Fish length (cm)** | **Acoustic tag model** | **DST** | **Capture region** | **Batch name** | **Release date** | **Tag recapture date** |
| --- | --- | --- | --- | --- | --- | --- | --- | --- |
| *C. auratus* | GGM01 | 56.4 | V13-1x | Y | Terschelling | TS1-22 | 2022-06-20 | 2023-11-30 |
| *C. labrosus* | TKM19 | 59.8 | V16-4x | N | Terschelling | TS4 | 2021-06-15 |  |
| *C. labrosus* | TKM96 | 48.8 | V13-1x | Y | Terschelling | TS1-22 | 2022-06-20 | 2023-07-24 |
| *C. labrosus* | TKM109 | 53.8 | V13-1x | Y | Terschelling | TS2-22 | 2022-06-21 | 2023-07-24 |
| *C. labrosus* | TKM110 | 55.2 | V13-1x | Y | Terschelling | TS2-22 | 2022-06-21 | 2024-03-29 |
| *C. labrosus* | TKM113 | 53.7 | V13-1x | Y | Terschelling | TS2-22 | 2022-06-21 |  |
| *C. labrosus* | TKM130 | 48.1 | V13-1x | Y | Terschelling | TS3-22 | 2022-06-22 |  |
| *C. labrosus* | TKM135 | 49.5 | V13-1x | Y | Terschelling | TS3-22 | 2022-06-22 | 2023-08-08 |
| *C. labrosus* | TKM167 | 49.5 | V13-1x | Y | Texel | TXN1-22 | 2022-07-26 | 2024-05-31 |

**Table S4 |** Yearly summary of detections for four example thickip (*C. labrosus*) and thinlip (*C. ramada*) grey mullet.

| Fish ID | Year | First detection | Last detection | N detections | N stations | Residence duration (days) |
| --- | --- | --- | --- | --- | --- | --- |
| TKM45 | 2021 | 2021-06-23 11:12:56 | 2021-07-05 22:55:14 | 79 | 6 | 13 |
| TKM45 | 2022 | 2022-07-21 19:24:58 | 2022-10-08 14:28:49 | 5 | 2 | 80 |
| TKM73 | 2021 | 2021-07-13 16:33:58 | 2021-09-27 04:36:57 | 84 | 5 | 77 |
| TKM73 | 2022 | 2022-06-08 13:03:59 | 2022-09-01 04:37:04 | 175 | 2 | 86 |
| TNM06 | 2021 | 2021-06-27 13:53:41 | 2021-09-26 04:32:46 | 464 | 19 | 92 |
| TNM06 | 2022 | 2022-05-18 05:15:48 | 2022-09-06 21:34:25 | 218 | 15 | 113 |
| TNM06 | 2023 | 2023-05-12 22:28:26 | 2023-10-10 16:43:00 | 469 | 18 | 152 |
| TNM08 | 2021 | 2021-07-01 19:19:02 | 2021-08-27 09:57:15 | 289 | 13 | 58 |
| TNM08 | 2022 | 2022-05-13 22:03:58 | 2022-08-17 08:35:16 | 85 | 9 | 96 |
| TNM08 | 2023 | 2023-05-17 02:19:35 | 2023-08-06 09:23:54 | 180 | 7 | 82 |
